# Supplementary material for: Survival status and predictors of mortality among low-birthweight neonates admitted to KMC units of five public hospitals in Ethiopia: Frailty survival regression model
Source: PLoS One. 2022 Nov 10;17(11):e0276291. doi: 10.1371/journal.pone.0276291 (PMC9648734; doi:10.1371/journal.pone.0276291)
Supplement: S1 Fig — (DOCX) [file pone.0276291.s001.docx]

**Proximate factors**

**Neonatal mortality**

**Maternal Factors:**

- History of abortion, history of still birth, maternal complications, gravidity, parity, age of mother’s

**Delivery Factors:**

- Place of death, place of birth, mode of delivery, assisted delivery

**Neonatal Factors:**

- Gestational age at birth, birthweight, birth size-for-gestational-age, sex of the newborn, age on admission, birth type, birth interval/order, neonatal complications,

**Newborn feeding practices:**

- Breastfeeding, complimentary feeding, initiation of first feed, hours after birth breastfeed initiated etc.

**Newborn care practices:** NICU admission, effective KMC, duration of SSC, Wash after birth, breastfeeding, etc.

**Socio-demographic factors**

**Household level:**

Monthly reported average income,

**Community level:**

Districts/sub-city, Health facility, altitude, residence

**Individual level:**

Marital status

Mother’s occupation

Mother’s education

Father’s occupation

Father’s education

Father’s age, Number of living children

S1 Figure: Conceptual framework in the study of survival status and predictors of neonatal mortality among LBW neonates in Oromia Regional State and Addis Ababa City, Ethiopia, 2019.
