# Supplementary material for: Survival status and predictors of mortality among low-birthweight neonates admitted to KMC units of five public hospitals in Ethiopia: Frailty survival regression model
Source: PLoS One. 2022 Nov 10;17(11):e0276291. doi: 10.1371/journal.pone.0276291 (PMC9648734; doi:10.1371/journal.pone.0276291)
Supplement: S3 Fig — (DOCX) [file pone.0276291.s003.docx]

**S3 Figure:** The Kaplan-Meier failure estimates compare time to death of neonate with categories of birthweight among neonates admitted to KMC unit, Ethiopia, 2017-2019.
