# Supplementary material for: Survival status and predictors of mortality among low-birthweight neonates admitted to KMC units of five public hospitals in Ethiopia: Frailty survival regression model
Source: PLoS One. 2022 Nov 10;17(11):e0276291. doi: 10.1371/journal.pone.0276291 (PMC9648734; doi:10.1371/journal.pone.0276291)
Supplement: S4 Fig — (DOCX) [file pone.0276291.s004.docx]

**S4 Figure: Kaplan-Meier failure estimates compare time to death of LBW newborns with categories of hours after birth first put the baby to the breast among LBW neonates admitted to KMC units of five public hospitals in Oromia Region and Addis Ababa City, Ethiopia, 2017 – 2019**
