# Supplementary material for: Survival status and predictors of mortality among low-birthweight neonates admitted to KMC units of five public hospitals in Ethiopia: Frailty survival regression model
Source: PLoS One. 2022 Nov 10;17(11):e0276291. doi: 10.1371/journal.pone.0276291 (PMC9648734; doi:10.1371/journal.pone.0276291)
Supplement: S5 Fig — (DOCX) [file pone.0276291.s005.docx]

S5 Figure: Graphical evaluation of the Weibull frailty (individual) regression model assumption by using the covariate birthweight-for-gestational-age for the study, Ethiopia, 2019.
