# Supplementary material for: Survival status and predictors of mortality among low-birthweight neonates admitted to KMC units of five public hospitals in Ethiopia: Frailty survival regression model
Source: PLoS One. 2022 Nov 10;17(11):e0276291. doi: 10.1371/journal.pone.0276291 (PMC9648734; doi:10.1371/journal.pone.0276291)
Supplement: S1 Table — (DOCX) [file pone.0276291.s006.docx]

S1 Table: Life table showing the cumulative probability of surviving stratified by birth size-for-gestational-age among LBW neonates admitted to KMC units of five Public Hospitals in Oromia region and Addis Ababa City, Ethiopia, 2017 – 2019.

| **Interval** | **Beg. Total** | **Deaths** | **Lost** | **Survival** | **Std. Error** | **95% CI** | |
| --- | --- | --- | --- | --- | --- | --- | --- |
| **Preterm-SGA** | | | | | | Lower | Upper |
| 0-3 | 323 | 33 | 3 | 89.74 | 0.02 | 85.87 | 92.59 |
| 3-6 | 287 | 41 | 5 | 76.80 | 0.02 | 71.77 | 81.06 |
| 6-9 | 241 | 17 | 8 | 71.29 | 0.03 | 65.96 | 75.95 |
| 9-12 | 216 | 7 | 13 | 68.91 | 0.03 | 63.47 | 73.72 |
| 12-15 | 196 | 3 | 11 | 67.83 | 0.03 | 62.33 | 72.70 |
| 15-18 | 182 | 2 | 20 | 67.04 | 0.03 | 61.49 | 71.97 |
| 18-21 | 160 | 1 | 12 | 66.60 | 0.03 | 61.03 | 71.57 |
| 21-24 | 147 | 3 | 5 | 65.22 | 0.03 | 59.53 | 70.31 |
| 24-27 | 139 | 1 | 2 | 64.75 | 0.03 | 59.02 | 69.88 |
| 27-30 | 136 | 7 | 129 | 58.41 | 0.03 | 51.48 | 64.70 |
| **Term-SGA** | | | | | | | |
| 0-3 | 165 | 26 | 0 | 84.24 | 0.03 | 77.73 | 88.99 |
| 3-6 | 139 | 7 | 0 | 80.00 | 0.03 | 73.04 | 85.34 |
| 6-9 | 132 | 7 | 6 | 75.66 | 0.03 | 68.34 | 81.52 |
| 9-12 | 119 | 2 | 5 | 74.36 | 0.03 | 66.93 | 80.36 |
| 12-15 | 112 | 3 | 6 | 72.31 | 0.04 | 64.72 | 78.54 |
| 15-18 | 103 | 1 | 1 | 71.61 | 0.04 | 63.96 | 77.92 |
| 18-21 | 101 | 0 | 3 | 71.61 | 0.04 | 63.96 | 77.92 |
| 21-24 | 98 | 2 | 2 | 70.13 | 0.04 | 62.35 | 76.60 |
| 24-27 | 94 | 2 | 1 | 68.63 | 0.04 | 60.74 | 75.27 |
| 27-30 | 91 | 11 | 80 | 53.83 | 0.05 | 43.76 | 62.87 |
| **AGA** | | | | | | | |
| 0-3 | 286 | 18 | 1 | 93.70 | 0.01 | 90.18 | 95.98 |
| 3-6 | 267 | 21 | 0 | 86.33 | 0.02 | 81.77 | 89.82 |
| 6-9 | 246 | 8 | 15 | 83.43 | 0.02 | 78.57 | 87.28 |
| 9-12 | 223 | 6 | 16 | 81.10 | 0.02 | 76.00 | 85.23 |
| 12-15 | 201 | 1 | 10 | 80.69 | 0.02 | 75.54 | 84.86 |
| 15-18 | 190 | 0 | 15 | 80.69 | 0.02 | 75.54 | 84.86 |
| 18-21 | 175 | 2 | 7 | 79.75 | 0.02 | 74.48 | 84.05 |
| 21-24 | 166 | 0 | 5 | 79.75 | 0.02 | 74.48 | 84.05 |
| 24-27 | 161 | 1 | 5 | 79.24 | 0.02 | 73.90 | 83.61 |
| 27-30 | 155 | 4 | 151 | 75.26 | 0.03 | 68.68 | 80.65 |
| **LGA** | | | | | | | |
| 0-3 | 34 | 1 | 0 | 97.06 | 0.03 | 80.90 | 99.58 |
| 3-6 | 33 | 1 | 1 | 94.07 | 0.04 | 78.32 | 98.48 |
| 6-9 | 31 | 1 | 2 | 90.94 | 0.05 | 74.45 | 96.99 |
| 9-12 | 28 | 2 | 1 | 84.32 | 0.06 | 66.28 | 93.18 |
| 12-15 | 25 | 0 | 1 | 84.32 | 0.06 | 66.28 | 93.18 |
| 15-18 | 24 | 0 | 1 | 84.32 | 0.06 | 66.28 | 93.18 |
| 27-30 | 23 | 0 | 23 | 84.32 | 0.06 | 66.28 | 93.18 |
