# Supplementary material for: Survival status and predictors of mortality among low-birthweight neonates admitted to KMC units of five public hospitals in Ethiopia: Frailty survival regression model
Source: PLoS One. 2022 Nov 10;17(11):e0276291. doi: 10.1371/journal.pone.0276291 (PMC9648734; doi:10.1371/journal.pone.0276291)
Supplement: S2 Table — (DOCX) [file pone.0276291.s007.docx]

S2 Table: The log likelihood of different parametric survival model along with their AIC and BIC

| Model | Frailty | Log likelihood | AIC | BIC |
| --- | --- | --- | --- | --- |
| Weibull | Without frailty | -190.55 | 433.09 | 536.1476 |
| **Weibull (PH)** | **Univariate (gamma)** | **-181.39** | **416.78** | **523.79** |
| Weibull (AFT | Univariate (gamma) | -181.49 | 416.88 | 523.89 |
| Weibull (PH) | Shared frailty (g) | -190.55 | 433.09 | 536.15 |
| Weibull (AFT) | Shared frailty (g) | -190.55 | 433.09 | 536.15 |
| Weibull (PH) | Shared frailty (IG) | -190.55 | 435.09 | 542.11 |
| Weibull (AFT) | Shared frailty (IG) | -190.55 | 435.09 | 542.11 |
| Loglogistic (PH) | Univariate (gamma) | -181.71 | 417.42 | 524.44 |
| Loglogistic (PH) | Univariate (IG) | -181.65 | 417.29 | 524.31 |
|  |  |  |  |  |
