# Supplementary material for: Survival status and predictors of mortality among low-birthweight neonates admitted to KMC units of five public hospitals in Ethiopia: Frailty survival regression model
Source: PLoS One. 2022 Nov 10;17(11):e0276291. doi: 10.1371/journal.pone.0276291 (PMC9648734; doi:10.1371/journal.pone.0276291)
Supplement: S3 Table — (DOCX) [file pone.0276291.s008.docx]

S3 Table: Bivariate frailty model showing CHRs and p-values in the study of predictors of mortality among LBW neonates admitted to KMC units of five public hospitals in Oromia Region and Addis Ababa City, Ethiopia.

| Socio-demographic, maternal and neonatal Characteristics | | | | | | | | |
| --- | --- | --- | --- | --- | --- | --- | --- | --- |
| Characteristics | Responses | CHR | P-value | Characteristics | Responses | CHR | P-value |  |
| Marital status | Single | 2.27 | 0.49 | NICU admission | No | 0.49 | 0.24 |  |
|  | Divorced | 1.91 | 0.26 |  |  |  |  |  |
|  | Married (ref) | 1.00 |  |  | Yes (ref.) | 1.00 |  |  |
| Mother’s education | No education | 0.89 | 0.99 | Maternal Complication | Yes | 1.13 | 0.24 |  |
|  | Primary | 0.65 | 0.96 |  | No (Ref.) | 1.00 |  |  |
|  | Secondary | 1.11 | 0.99 | Mode of delivery | Assisted vaginal delivery | 1.03 | 0.95 |  |
|  | Above secondary (ref.) | 1.00 |  |  | Caesarean section | 0.82 | 0.35 |  |
| Mother’s occupation | Agriculture | 1.82 | 0.87 |  | SVD (Ref.) | 1.00 |  |  |
|  | House wife | 1.45 | 0.201 | Birth interval | < 3 Years | 1.29 | 0.27 |  |
|  | Sales and services | 0.86 | 0.706 |  | ≥ 3 Years (Ref.) | 1.00 |  |  |
|  | Skilled manual | 1.46 | 0.336 | Multiple birth | Twins | 0.89 | 0.41 |  |
|  | Unemployed | 2.89 | 0.014 |  | Triplets | 1.15 | 0.76 |  |
|  | Unskilled manual | 1.57 | 0.240 |  | Singleton (Ref.) | 1.00 |  |  |
|  | Professional work (ref.) | 1.00 |  | Sex of the baby | Male | 1.35 | 0.09 |  |
| Father’s age (years) | < 25 | 0.91 | 0.69 |  | Female (Ref.) | 1.00 |  |  |
|  | 25 to 29 | 0.96 | 0.78 | Birth weight | <1000 grams | 24.26 | 0.043 |  |
|  | 30 to 34 | 0.58 | 0.003 |  | 1000 to 1499 grams | 21.63 | 0.001 |  |
|  | ≥ 35 (ref.) | 1.00 |  |  | 1500 to <20000 (Ref.) | 1.00 |  |  |
| Father’s education | No Education | 2.22 | 0.11 | Being born preterm | Yes | 3.05 | 0.24 |  |
|  | Primary (grade 1-8) | 1.90 | 0.028 |  | No (ref.) | 1.00 |  |  |
|  | Secondary (grade 9-12) | 1.56 | 0.122 | Birth size-for-gestational-age | Preterm-SGA | 2.23 | 0.75 |  |
|  | Above secondary (ref.) | 1.00 |  |  | Term-SGA | 23.22 | 0.64 |  |
| Avg. family monthly income | | 0.99 | 0.98 |  | LGA (Ref.) | 0.07 | 0.208 |  |
| Number of living children | 2 to 4 | 0.83 | 0.96 |  | AGA (ref.) | 1.00 |  |  |
|  | >=5 | 1.44 | 0.24 | Neonatal complication | Yes | 345.13 | 0.001 |  |
|  | < 2 (ref.) | 1.00 |  |  | No (Ref.) | 1.00 |  |  |
| Mother’s age at birth | < 25 years | 3.27 | 0.18 |  |  |  |  |  |
|  | 25 to 29 years | 3.96 | 0.42 | Wash immediately after birth | Yes | 88.64 | 0.001 |  |
|  | ≥35 years | 2.91 | 0.25 |  | No (ref.) | 1.00 |  |  |
|  | 30 to 34 years (ref) | 1.00 |  | Hours after birth first put the baby to the breast | BF not yet initiated | 36.87 | 0.001 |  |
| Parity | Primiparous | 0.97 | 0.82 |  | Initiated 1 to <= 24 hrs | 0.58 | 0.146 |  |
|  | Grand multi-parous (5+) | 1.49 | 0.54 |  | Initiated after 24 hrs | 10.93 | 0.001 |  |
|  | Multi-parous (2-4)(ref.) | 1.00 |  |  | BF initiated < 1 hrs (ref.) | 1.00 |  |  |
| History of still birth | Yes | 1.05 | 0.86 | Effective KMC during initiation | No | 530.74 | 0.001 |  |
|  | No (ref.) | 1.00 |  |  | Yes (ref.) | 1.00 |  |  |
| History of abortion | Yes | 2.13 | 0.08 | Effective KMC during discharge | No | 13.19 | 0.001 |  |
|  | No (Ref.) | 1.00 |  |  | Yes (ref.) | 1.00 |  |  |
| Gravidity | Primi-gravida | 1.31 | 0.05 |  |  | | |  |
|  | Grand multi-gravida (V+) | 0.25 | 0.027 |  |  |  |  |  |
|  | Multi-gravida (ii-iv)(Ref.) | 1.00 |  |  |  |  |  |  |
