# Supplementary material for: Survival status and predictors of mortality among low-birthweight neonates admitted to KMC units of five public hospitals in Ethiopia: Frailty survival regression model
Source: PLoS One. 2022 Nov 10;17(11):e0276291. doi: 10.1371/journal.pone.0276291 (PMC9648734; doi:10.1371/journal.pone.0276291)
Supplement: Attachment — Submitted filename: PONE-D_21-16170R.pdf [file pone.0276291.s010.pdf]

Survival status and predictors of mortality among low -birth-weight neonates admitted to KMC units of five public hospitals in Ethiopia: frailty survival regression model

Manuscript Number: PONE-D-21-16170R

Comments to Authors and Editor

General comments

The authors attempt to determine the incidence of neonatal mortality rates and identify its predictors among LBW neonates born or treated at hospitals using frailty regression model. The study looks interesting; however, my main concern is the clarity of the presentation from methodological point of view. I think most readers would find it very difficult to implement the described approach in other data sets. The manuscript would be more strengthened by revising the methodology and results to make clear understanding.

Parametric frailty regression model

I couldn't find anywhere in the manuscript how to address the problem of heterogeneity in a population resulting from unobserved covariates. I prefer more justification to use frailty models in this setting, then readers can apply same analytical framework to their study. I strongly recommend avoiding mathematical notation as there is no implication for these notations in the result discussion.

Model building

Model building is always a risky business. Adding significant number of exposures, confounders and predictors looks more challenging for appropriate model building. I strongly suggest Authors to go through the scientific process of variables selection (lasso) in the model building strategy.

Presentation

The manuscript is overly long with a lot of redundancies. In this circumstance, I strongly recommend making shorter with clear message to the audience about mortality among LBW new-borns.
