# Supplementary material for: Survival status and predictors of mortality among low-birthweight neonates admitted to KMC units of five public hospitals in Ethiopia: Frailty survival regression model
Source: PLoS One. 2022 Nov 10;17(11):e0276291. doi: 10.1371/journal.pone.0276291 (PMC9648734; doi:10.1371/journal.pone.0276291)
Supplement: Attachment — Submitted filename: Response to reviewers.docx [file pone.0276291.s011.docx]

Manuscript Number: PONE-D-21-16170R

Response to Reviewers

Dear Dr. Funmilola M. OlaOlorun,
Academic Editor,

PLOS ONE,

Thank you for giving us the opportunity to submit a revised draft of the manuscript “Survival status and predictors of mortality among low -birth-weight neonates admitted to KMC units of five public hospitals in Ethiopia: frailty survival regression model” for publication in the PLOS ONE Journal. We appreciate you and the reviewers for your precious time in reviewing our paper and providing valuable comments. It was your valuable and insightful comments that led to possible improvements in the current version. The authors have carefully considered the comments and tried our best to address every one of them. We hope the manuscript after careful revisions meet your high standards. The authors welcome further constructive comments if any. Below we provide the point-by-point responses. Please see, in blue, for a point-by-point response to the reviewers. All modifications in the manuscript have been highlighted in track changes.

**Responses to points raised by editors**

1. *Please ensure that your manuscript meets PLOS ONE's style requirements, including those for file naming*.

We tried to prepare the manuscript according to PLOS ONE's style requirements. If any, we are ready to revise it again.

2. *Please provide additional details regarding participant consent. In the ethics statement in the Methods and online submission information, please ensure that you have specified whether consent was informed.*

All respondents were informed about the study procedures and the interview was conducted after obtaining their informed consent. The participants were assured of confidentiality as they participate in the investigation.

3. *Please include a separate caption for each figure in your manuscript.*

Accepted and corrected

4. *In your Data Availability statement, you have not specified where the minimal data set underlying the results described in your manuscript can be found. PLOS defines a study's minimal data set as the underlying data used to reach the conclusions drawn in the manuscript and any additional data required to replicate the reported study findings in their entirety. All PLOS journals require that the minimal data set be made fully available.*

The minimum data set is fully available within the manuscript.

**Responds to the reviewer’s comments:**

Thank you Dr. Shahid Ullah for your deep review of the paper

**Reviewer #2:**

**Response to comment:** *The authors attempt to determine the incidence of neonatal mortality rates and identify its predictors among LBW neonates born or treated at hospitals using frailty regression model.*

*The study looks interesting; however, my main concern is the clarity of the presentation from the methodological point of view. I think most readers would find it very difficult to implement the described approach in other data sets. The manuscript would be more strengthened by revising the methodology and results to make a clear understanding.*

Thank you for the suggestion. We have re-written this part according to the Reviewer’s comments. Considering the Reviewer’s suggestion, we add a detailed account of many of the methods parts of the manuscript.

**Reviewer #2:**

**Response to comment:** *I could not find anywhere in the manuscript how to address the problem of heterogeneity in a population resulting from unobserved covariates. I prefer more justification to use frailty models in this setting, then readers can apply same analytical framework to their study. I strongly recommend avoiding mathematical notation, as there is no implication for these notations in the result discussion*.

We are very sorry for not including the justification to use the frailty model in this setting. Considering the Reviewer’s suggestion, we add a detailed account of the justification to use the frailty model. In this study, neonates that were treated in the same hospital might share similar facilities, physicians, midwives, and other neonatal care practices. They might also share some similarities that were not measured as covariates and varied by the hospital. These hospital effects might create dependence between the study outcomes at each hospital and lead the failure times to correlate. Therefore, to account for this correlation between individuals within clusters (in this case hospitals), a random-effects survival model, parametric shared frailty model, was employed to find out independent predictors of neonatal mortality. Here, frailty describes hospital-to-hospital variations that were not explained by observed covariates. The shared frailty model assumes that individuals in a subgroup (in the same hospital) share the same frailty (α), but frailty from group to group may differ. A one-parameter gamma frailty distribution with mean 1 and variance θ, and Weibull distribution for the baseline hazard, was employed.

**Reviewer #2:**

**Response to comment:** *Model building is always a risky business. Adding significant number of exposures, confounders and predictors looks more challenging for appropriate model building. I strongly suggest Authors to go through the scientific process of variables selection (lasso) in the model building strategy*.

Thank you for the suggestion. I know you are expertise in the area. I am really very satisfied with this comment and it adds new knowledge for me. As you know, most recommend the lasso method when there is high dimensional data (the number of variables is many, specifically if the number of independent variables is much greater than the sample size). (This is from my reading after your review). We have few variables and large sample size (n=808) in this manuscript. We have greater than thirty (30) respondents/or number of cases for one variable. Twenty-five (25) variables were entered for the bivariate analysis. From these, only fifteen (15) were significant in the bivariate analysis and were entered for multivariable analysis. To prevent overfitting due to either collinearity of the covariates or high dimensionality, we did also a multicollinearity check through VIF and there were no variables that were correlated to each other. We hope the results will be nearly similar if done with other methods of variable selection. As you mentioned, variable selection for survival data analysis poses many challenges because of the complicated data structure. As to my reading, the lasso method mainly works for linear, logit, probit, and Poisson models. I couldn’t find a lasso in STATA for the frailty model. I hope you will understand the difficulties I encountered. If you have a further concern please let me know before a decision.

**Reviewer #2:**

**Response to comment:** *The manuscript is overly long with many redundancies. In this circumstance, I strongly recommend making shorter with clear message to the audience about mortality among LBW newborns.*

Thank you for the suggestion. We have tried to avoid the redundancies and re-written the manuscript according to the Reviewer’s comments

We tried our best to improve the manuscript and made some changes in the manuscript. We appreciate for Editors/Reviewers’ warm work earnestly, and hope that the correction will meet with approval.

Once again, thank you very much for your comments and suggestions.
