# Supplementary material for: Survival status and predictors of mortality among low-birthweight neonates admitted to KMC units of five public hospitals in Ethiopia: Frailty survival regression model
Source: PLoS One. 2022 Nov 10;17(11):e0276291. doi: 10.1371/journal.pone.0276291 (PMC9648734; doi:10.1371/journal.pone.0276291)
Supplement: Attachment — Submitted filename: Review Comments.docx [file pone.0276291.s012.docx]

**Comments**

**Introduction**

The rational of the research is not clear and not novel. Many studies were conducted on this area that accounted multiple predictors. What makes this study unique?

**Methods**

It is not clear how the sample size is determined. How do you know the sample size used is sufficient enough for the analysis method you employed?

Sampling method; what do you mean by consecutive? Do you mean you included everyone during the study period? Is that a kind of cluster sampling?

You do not need “variable of the study” session

Outcome variable definition seems binary. But for this research it is time to event. So, correct your outcome variable definition.

Data collection instrument and process: How did you track those who were discharged before 28 days? How frequent you visited the new-born after discharge?

How could you treated twins and triplets as independent? I suggest you to take one among the twin or triplets.

Data analysis: the choice of the statistical model (random effect survival) seems strange. Since the number of clusters (hospitals) are very few frailty may not be appropriate.

**Results**

The descriptive results were not presented based survival data analysis form. Where is information related to the life table? Table 1-3 should be changed as per the comment.

Person time of observation (the time a risk) has to be the first information presented in this part.

All the percentages should take in to account person time of observation.

The survival curve is not correctly presented (Figure 3). This should be modified and replaced with the standard presentation of the survival curve.

Table 5 should be modified and the number of event for each category of the variables should be presented.

Discussion

The first paragraph should present all the necessary findings of the paper.
