# Supplementary material for: Survival status and predictors of mortality among low-birthweight neonates admitted to KMC units of five public hospitals in Ethiopia: Frailty survival regression model
Source: PLoS One. 2022 Nov 10;17(11):e0276291. doi: 10.1371/journal.pone.0276291 (PMC9648734; doi:10.1371/journal.pone.0276291)
Supplement: Attachment — Submitted filename: Response to reviewers.docx [file pone.0276291.s013.docx]

Manuscript Number: PONE-D-21-16170R

Response to Reviewers

Dear Dr. Funmilola M. OlaOlorun,
Academic Editor,

PLOS ONE,

Thank you for allowing us the opportunity to submit a revised draft of the manuscript “Survival status and predictors of mortality among low -birth-weight neonates admitted to KMC units of five public hospitals in Ethiopia: frailty survival regression model” for publication in the PLOS ONE Journal. We appreciate the time and effort that the reviewers have dedicated to providing valuable feedback on our manuscript, and are grateful for their constructive criticism. It was your valuable and insightful comments that led to possible improvements in the current version. We have now revised the manuscript to reflect most of the suggestions provided by the reviewers, and feel that it is significantly improved. We hope the manuscript after careful revisions meet your high standards. The authors welcome further constructive comments if any. Below we provide the point-by-point responses. Please see, in Blue, for a point-by-point response to the reviewers. All modifications in the manuscript have been highlighted in track changes.

**Responses to points raised by editors**

1. *The reviewers of the revised version of this manuscript have expressed concerns about your choice of a frailty model, and the reproducibility of the methods of this research. If you choose to revise further, please ensure you are intentional about describing and justifying your methodological approach. In addition, more can be done to improve the clarity and readability of the manuscript*

Thank you for your suggestion. The notion of frailty provides a convenient way to introduce random effects, association, and unobserved heterogeneity into models for survival data. In its simplest form, frailty is an unobserved random factor that modifies multiplicatively the hazard function of an individual or a group or cluster of individuals. The goal is to estimate a “frailty” parameter that “absorbs” the effects of these unmeasured risk factors/covariates. In statistical terms, a frailty model is a random effect model for time-to-event data, where the random effect (the frailty) has a multiplicative effect on the baseline hazard function. It extend Cox proportional hazards (PH) model by introducing unobserved “frailties” to the model.

In our study, there are unobserved covariates (e.g. nutritional status of the mother during pregnancy, weight gain during pregnacy) or unobservable heterogeneity, caused by different sources. We considered a random effects survival (frailty) model to take into account these unobserved effects. Initially, we planned to fit the shared frailty model to account for cluster effects or variations between clusters (hospitals) and to incorporate correlation within the same clusters since the same cluster shares the same random effects. We treat hospitals as clusters. Due to very few clusters, shared (clustered survival analysis) may not be appropriate, as the reviewers also suggested. We checked the best-fitting model using the information criterion (AIC and BIC). Based on these criteria, the univariate/ non-shared/ or individual frailty model is the best-fitting model among others. Frailty models for univariate data have been used to account for heterogeneous times-to-failure among individuals. Usually, it is thought of as unmeasured risk factors. If there are unmeasured or unobserved “frailties,” the hazard rate will not only be a function of the covariates, but also a function of the frailties. A frailty model in the univariate case introduces an unobservable multiplicative effect α on the hazard, so that conditional on the frailty: h(t|α) = αh(t).

h(t_i_|α_i_) = α_i_h(t_i_) = α_i_h_0_(t_i_) exp(X^t^_i_ β)	……… (1)

Where α is some random positive quantity assumed to have mean one and variance θ. Those individuals who possess α > 1 are said to be more frail for reasons left unexplained by the covariates and will have an increased risk of failure. Conversely, those individuals with α < 1 are less frail and will tend to survive longer all else being equal (i.e., given a certain covariate pattern). Since α is a multiplicative effect, it is easy to see from (1) how one can think of frailty as representing the cumulative effect of one or more omitted covariates. If α was not significant in this study, the model can be lowered into a fixed effects model whether parametric (e.g. Weibull regression) or semi-parametric (e.g. Cox), or others. Since the LR test of theta (θ) = 0 is significant ( P-value = 0.001), suggesting that the frailty element contributes to the model (clue towards the existence of undetected heterogeneity). Hence, the individual frailty regression model is the appropriate model for this study. The manuscript is also revised by considering this change.

**Responds to the reviewer’s comments:**

Thank you Reviewer #3 for your deep review of the paper

**Reviewer #3:**

**Response to comment:** *The rational of the research is not clear and not novel. Many studies were conducted on this area that accounted multiple predictors. What makes this study unique?*

First, thank you for the question.

This study is conducted among low birthweight (LBW) babies (born <2000 grams). Many previous studies in Ethiopia report the mortality rates among LBW babies (not stratified rate). The main causes of LBW are preterm birth and growth restriction (SGA). Although preterm birth and SGA have common causes they have also different causes. Merging and reporting the mortality rate as LBW might hinder prevention mechanisms. So reporting the stratified incidence rates of mortality may advance the prevention mechanisms of LBW. In Ethiopia, if a baby born <1000 grams and/or <28 weeks of gestation is treated as abortion irrespective of viability. In this study as part of a large KMC implementation research project, we admitted all these newborns, if viable, to the KMC unit. Many babies who were <1000 grams and even at 24 weeks of gestation survived. In this study, we also evaluated effective KMC can advance the survival of very small babies in Ethiopia. This has an operational policy impact on the Ethiopian population.

**Reviewer #3:**

**Methods sections:**

**Response to comment:** *It is not clear how the sample size is determined. How do you know the sample size used is sufficient enough for the analysis method you employed?*

Now, the sample size calculated to estimate the incidence rate of mortality was checked whether it is enough or not to identify predictors of mortality. Using a two-sample comparison of survivor functions (Log-rank test, Freedman method), the number of events (E) that could be needed was calculated by: E = ((Z_α/2_ + Z_β_)^^2^)/((ln(HR))^^2^ pq) in STATA software. Using Freedman principles q = rate of an event is equal to p = survival probability rate (proportional allocations, p = q, the median survival = 50%). To calculate the total number of events (E) that could be needed to compare the two groups (exposed and unexposed/1:1 ratio), we take α = 0.05 and β = 0.20. With these values of α and β, Z_α/2_ = 1.96 and Z_β_ = 0.84, and taking the HR 1.65 from previous study, and 10% withdrawal, the number of events (E) becomes 132. Then, the total number of participants needed (n) for the survival study was calculated by using n = E/Pr(event), and it becomes 407 by considering 1.5 clustering effect. We now mention these considerations in the methods section.

**Reviewer #3:**

**Response to comment:** *Sampling method; what do you mean by consecutive? Do you mean you included everyone during the study period? Is that a kind of cluster sampling?*

Thank you for the question. Yes, it is to mean everyone during the study period was included (i.e. cluster sampling). We now mention these considerations in the methods section.

*You do not need “variable of the study” session*

*Outcome variable definition seems binary. But for this research it is time to event. So, correct your outcome variable definition.*

Thank you for your suggestion. All the given suggestions are accepted and the manuscript is revised according to the reviewer’s comments.

**Reviewer #3:**

**Response to comment:** *Data collection instrument and process: How did you track those who were discharged before 28 days? How frequent you visited the new-born after discharge?*

Thank you for your suggestion. There were follow-up home visits at seven days of life, seven days post-discharge, and 29 days of life to track information if the baby was discharged before 28 days of life. We now mention these considerations in the methods section (data collection instrument and process).

**Reviewer #3:**

**Response to comment:**

*How could you treated twins and triplets as independent? I suggest you to take one among the twin or triplets.*

Thank you for the question. It can be done as you suggested. But, from a clinical point of view and from many other previous studies, the risk of mortality is increased with the number of births (singleton vs multiple births). Taking singleton and one from twins or triplets may underestimate the outcome of interest (mortality). So it is better to take all types of births to estimate the true incidence rate of mortality.

**Reviewer #3:**

*Data analysis: the choice of the statistical model (random effect survival) seems strange. Since the number of clusters (hospitals) are very few frailty may not be appropriate.*

Thank you for your suggestion. As you rightly said the number of clusters are very few (five), and the estimate may be unstable in case of shared frailty. We now fit and compare around nine (9) parametric survival models including the Weibull regression model and shared frailty regression model to see the well-fitted model for the data. Using the AIC and BIC, the individual/univariate frailty regression model (unshared) is the best-fitting model. Look S2 Table (in the supplementary materials). We now mention these considerations in the results section.

**Reviewer #3:**

Results

*The descriptive results were not presented based survival data analysis form. Where is information related to the life table? Table 1-3 should be changed as per the comment.
Person time of observation (the time a risk) has to be the first information presented in this part. All the percentages should take in to account person time of observation.*

Thank you for your suggestion. All the comments are accepted and incorporated into the document. Life table showing the overall cumulative probability of surviving (Table 5) and birth size-for-gestational-age specific cumulative probability of surviving (S1 Table) were produced. The total time at risk is placed as you suggested and Tables 1-3 are also modified as you suggested (neonatal days of observation were taken into account to compute the incidence rate of mortality).

**Reviewer #3:**

*The survival curve is not correctly presented (Figure 3). This should be modified and replaced with the standard presentation of the survival curve.*

Thank you for your suggestion. The KM survival estimate is modified and replaced with the standard presentation of the survival curve (look at Fig. 3 and S2 Fig.)

**Reviewer #3:**

*Table 5 should be modified and the number of event for each category of the variables should be presented.*

Thank you for your suggestion. Revised as you suggested, the number of events and censured (with %) are now included. We now mention these considerations in Table 7.

**Reviewer #3:**

Discussion
The first paragraph should present all the necessary findings of the paper.

Thank you for your suggestion. We now mention these considerations in the discussion section.

Thank you Reviewer #4 for your deep review of the paper

**Reviewer #4:**

*This is important work, especially for Ethiopia, and the authors have made great efforts to address comments in a prior review. While the conclusions are not really new, this may be important in the national policy setting context. A few issues remain:
1. Babies transferred to an outside facility after 3 days of life were excluded. Since there is a sizable number of babies fitting this criterion, and no information is provided on the characteristics of these excluded babies vs those in the analysis, it is unclear what effect this has on the results and conclusions.*

Thank you for your suggestion. We checked the differences for baseline characteristics of babies included and excluded (like transferred to an outside facility after 3 days of life). According to the statistical test made, there were no statistically significant differences between babies who were included and excluded in this analysis. We now mention these considerations in the results section.
**Reviewer #4:**

*2. The sample size calculation approach is poorly explained. Is it based on the precision/width of the confidence interval for an assumed rate of NMR? Please clarify.*
Thank you for your suggestion.

However, we included everyone during the study period (n = 808), and the minimum required sample size was calculated using one group dichotomous outcome variable (mortality: died, censured) using STATA statistical package using the following formula:

$$n=\frac{\left( Z\alpha\sqrt{po \left( 1-Po \right)} +Z\beta\sqrt{P1 (1-P1} \right)^^{2}}{\left( P1-Po \right)^{2}}$$

Assumptions used for the calculation were: neonatal mortality rate of 28% based on previous estimates (P_o_); we want to sample a group large enough to detect an incidence rate of 34% (i.e. H_1_: P_1_ = 0.34) if that is the real, true incidence. We set the two-sided α at 0.05 and want a power of 0.80 to detect the difference in incidence setting the rate of deaths of neonates in the area as high (P_1_ = 34%) to make the sample size large enough to detect the true incidence. And 10% lost-to-follow-up; and 1.5 for clustering effect. The sample size was also determined by considering the model that was fitted. We now mention these considerations in the methods section.

**Reviewer #4:**

*3. The model selection approach may not be optimal. First, basing covariate selection strictly on empirical bivariate analysis results by definition cannot identify hidden confounders and higher level interactions. Second, using AIC is more appropriate for developing a prediction model and not necessarily for identifying causal pathways or markers for the outcome.*
Thank you for your suggestion. To evaluate the application of mixed-effects survival (frailty) model, ln(-ln(S(t))) was plotted against ln(t). The curves in the graph seem to have nearly the same slope (i.e., are parallel, have the same p) signifying that the PH (and thus the AFT) assumptions hold. This signals the effect of exposure constantly rises over time and proposing the Weibull individual frailty regression assumptions is evenhanded. To select the best-fitting model (post estimation), the AIC and BIC were used. In this criteria, the preferred model is the one with the lowest value of the AIC or BIC. Consequently, the Weibull gamma frailty (univariate) model in the PH metric was the best well-fitted model (S2 Table). We started with the classical bivariate analysis and then stepwise regression, for choosing predictor variables from a large set. Now, we considered AIC for model selection/post estimation (prediction model), not for covariate selection as you suggested. We now mention these considerations in the methods section.

**Reviewer #4:**

*4. Presentation of stratified/subgroup analysis should be ideally preceded by and conditional on a significant interaction test (between SGA and prematurity in this instance).*
Thank you for your suggestion. Yes, the comment is accepted and the manuscript is revised as you suggested (Table 7 also includes the subgroup analysis).

**Reviewer #4:**

*5. It is unclear if CHR (table 5) is ever defined.*

Sorry, it is to mean crude hazard ratios (CHRs)
**Reviewer #4:**

*6. The authors state that all the relevant data is available within the manuscript, but I think the journal is asking for individual and not aggregate level data. I did not see the former included*.

Thank you for your suggestion. Now all the relevant required data are within the manuscript. If the journal needs the database, we can upload it or can be accessed it upon request.

We tried our best to improve the manuscript and made many changes in the manuscript. We appreciate for Editors/Reviewers’ warm work earnestly and hope that the correction will meet with approval.

Once again, thank you very much for your comments and suggestions.
