# Supplementary material for: Survival status and predictors of mortality among low-birthweight neonates admitted to KMC units of five public hospitals in Ethiopia: Frailty survival regression model
Source: PLoS One. 2022 Nov 10;17(11):e0276291. doi: 10.1371/journal.pone.0276291 (PMC9648734; doi:10.1371/journal.pone.0276291)
Supplement: Attachment — Submitted filename: Response to reviewers.docx [file pone.0276291.s014.docx]

Manuscript Number: PONE-D-21-16170R

Response to Reviewers

Dear Dr. Funmilola M. OlaOlorun,
Academic Editor,

PLOS ONE,

Thank you for allowing us the opportunity to submit a revised draft of the manuscript “Survival status and predictors of mortality among low -birth-weight neonates admitted to KMC units of five public hospitals in Ethiopia: frailty survival regression model” for publication in the PLOS ONE Journal. We appreciate the time and effort that the reviewers have dedicated to providing valuable feedback on our manuscript, and are grateful for their constructive criticism. It was your valuable and insightful comments that led to possible improvements in the current version. We have now revised the manuscript to reflect most of the suggestions provided by the reviewers, and feel that it is significantly improved. We hope the manuscript after careful revisions meet your high standards. The authors welcome further constructive comments if any. Below we provide the point-by-point responses. Please see, in Blue, for a point-by-point response to the reviewers. All modifications in the manuscript have been highlighted in track changes.

**Responses to points raised by editors**

1. *Please review your reference list to ensure that it is complete and correct. If you have cited papers that have been retracted, please include the rationale for doing so in the manuscript text, or remove these references and replace them with relevant current references. Any changes to the reference list should be mentioned in the rebuttal letter that accompanies your revised manuscript. If you need to cite a retracted article, indicate the article’s retracted status in the References list and also include a citation and full reference for the retraction notice.*

Thank you for your suggestion. The reference list is checked, and it is complete and correct.

2. *We thank the authors for their efforts in responding to the comments raised by the expert reviewers. Reviewer 3 would like to see additional minor edits before we can consider this manuscript for publication. Additionally, it would be great if the authors can read through again to edit grammatical and typographical errors since the journal does not offer any copy editing. Thank you.*

Thank you for your suggestion. We made some grammatical and typographical edits.

**Responds to the reviewer’s comments:**

Thank you again both Reviewers

**Reviewer #3 and #4**

**Response to comment:** *Have the authors made all data underlying the findings in their manuscript fully available?*

We now uploaded the data set in Excell format.

**Reviewer #3:**

**Methods sections:**

**Response to comment:** *The authors attempted to address the comments. However, I still have the following concerns;*

1. *The samplse size you defined (formula) should match the methods of analysis you used. Sample size for surviavl analysis is different from the sample size for proportion. Remove the formula and keep the text explanation.*

Thank you for your suggestion. The sample size calculation formula is deleted. We now mention these considerations in the methods section

2. *Table 5 is not important to be presented in the document. KM can provide similar information instead of life table. So, remove table 5 from the document.*

Thank you for your suggestion. Table 5 is removed as you suggested. We now mention these considerations in the results section

We tried our best to improve the manuscript and made many changes in the manuscript. We appreciate for Editors/Reviewers’ warm work earnestly and hope that the correction will meet with approval.

Once again, thank you very much for your comments and suggestions.
